# Fatty Acid Composition of Salami Made by Meat from Different Commercial Categories of Indigenous Dairy Cattle

**DOI:** 10.3390/ani11041060

**Published:** 2021-04-08

**Authors:** Marco Alabiso, Giuseppe Maniaci, Cristina Giosuè, Antonino Di Grigoli, Adriana Bonanno

**Affiliations:** 1 Dipartimento Scienze Agrarie, Alimentari e Forestali, University of Palermo, Viale delle Scienze, 90128 Palermo, Italy; marco.alabiso@unipa.it (M.A.); antonino.digrigoli@unipa.it (A.D.G.); adriana.bonanno@unipa.it (A.B.); 2 Institute for Anthropic Impacts and Sustainability in the Marine Environment, National Council of Research (IAS-CNR), 90128 Palermo, Italy; cristina.giosue@ias.cnr.it

**Keywords:** cinisara breed, beef, cured meat, fat, fermented sausage

## Abstract

**Simple Summary:**

The Cinisara is a Sicilian breed raised on pasture to produce the Caciocavallo Palermitano cheese. Even if it is penalized by competition with meat breeds, characterized by higher growth rate and yield, the production of fresh meat represents a considerable added value for the smallfarms. The meat of Cinisara is not appreciated, despite having a high content of iron, vitamin E, conjugated linoleic acid (CLA), and low content of lipids and cholesterol, above all due to incorrect management of the supply chain phases that negatively affects the quality of the final product. Alternative production such as bresaola and salami could contribute to the enhancement of Cinisara meat. The present study investigated the fatty acid profile of salamis produced by processing the meat of young bulls and adult cows with the addition of lard pork to provide additional information to that reported in a previous experiment on physicochemical and sensory properties. The results suggest the possibility of producing cured meats with Cinisara meat, even if the addition of pork lard mitigates some favorable effects deriving from the livestock system of this breed, based on grazing. Further studies should be conducted to investigate the possibility of making cured meats with beef only.

**Abstract:**

In autochthonous dairy cattle farms, the production of salami could represent an alternative commercial opportunity. Therefore, a study was carried out to investigate the fatty acid (FA) composition of salami made using the meat from grazing (GB) or housed (HB) young bulls and grazing adult cows (AC) of Cinisara breed. The products were manufactured by adding 20% of pork lard. Animal category influenced the FA composition, although the addition of lard mitigated the differences found in fresh meat. The salami from GB showed higher polyunsaturated FA content (*p* ≤ 0.01) and, in particular, a higher level of linoleic acid (*p* ≤ 0.05), than from other animal categories. Salami made from AC meat showed lower polyunsaturated/saturated FA ratio (*p* ≤ 0.05), but a better *n-6*/*n-3* ratio compared to HB (*p* ≤ 0.05), due to the lower content of linoleic acid. Multivariate analysis showed an important influence of animal category on FA composition due to age, feeding system and meat fat content of animals, despite the addition of lard.

## 1. Introduction

Cinisara cow, a Sicilian autochthonous dairy breed, is traditionally reared with a feeding system based on the prevalent exploitation of natural pasture, and its milk is used to produce the Caciocavallo Palermitano cheese [1,2,3]. The manufacture of processed products, such as salami and bresaola, an alternative to fresh meat, could increase the economic profit of farms, diversifying the offer in the market [4,5,6,7]; in particular, some cuts of the posterior quarter (semimembranosus, semitendinosus and quadriceps femorismuscles) and the anterior quarter (brachial biceps muscle) could be used for the production of bresaola and the rest of the meat for the production of salami.

In recent years, consumers’ interest in products rich in protein and low in lipid has increased, promoting the consumption of alternative meat to pork. Traditionally, the salami is made with meat and fat of pork [8], and shows high-fat content [9]. Therefore, the reformulation of meat-based products is directed to the promotion of consumer health by reducing lipid content and improving its fatty acid (FA) profiles [10]. In particular, lipid reformulation is based on the use of low-fat meat and/or the replacing of the fat with one having better healthy characteristics. Interest in cured, fermented and dried products of other livestock animals, such as cattle, donkeys, mutton and poultry has greatly increased, representing alternative products in the market [11,12,13].

During fermentation and ripening of salami, several biochemical, microbiological, physical and sensorial changes occur in meat under defined conditions of temperature and relative humidity (RH). Specifically, carbohydrates, proteins and lipids are the main substrates of these reactions. Endogenous muscular enzymes (cathepsins) may determine proteolysis and lipolysis at the value of pH found in fermented sausages, as well as the microorganisms’ enzymes [14,15,16]. Lipolytic phenomena, with the release of FA and carbonyl compounds, and proteolytic phenomena, with the release of non-protein nitrogen compounds, are important for the development of characteristic taste and flavor of the final product [17,18].

In meat, spontaneous fermentation is associated with the biochemical activity of indigenous lactic acid bacteria (LAB). This process is technically easy and inexpensive but determines a certain variability in quality products and increases the risk of contamination from pathogenic microorganisms [19]. Controlled fermentation is an alternative, and involves the use of selected bacteria (living or resting) as starters, developing the desired metabolic activity in the meat. Further, different strains of indigenous LAB from meat are also isolated and used as commercial starters to made salami [20,21]. Consequently, the starter cultures influence the lipolytic processes, even if their effects on the FA composition are not clear. For example, higher contents of saturated FA and linoleic acid were found in Pastirma (Turkish dry-cured sausage) made using starter cultures [22], while no effect was detected in Tunisian sausage [23].

The breed, the age, the gender and the feeding system can influence the FA composition of fresh meat [24,25,26,27,28]. As also found in bresaola, grazing improves health and organoleptic properties of processed meat products compared to products obtained with the meat of animals reared in confined systems, positively influencing the FA profiles [4]. Indeed, different studies showed the use of pasture increases the percentage of *n-3* and conjugated linoleic acid (CLA) in products, which are FA with beneficial effects on human health [27]. Otherwise, the products from animals that are concentrate-fed show generally high level of *n-6*, in particular linoleic acid, negatively affecting their healthy quality [29].

The diet administered to the animal is one of the most important factors influencing the composition of production in cattle and sheep. This effect is due to specific compounds, including FA, which is transferred directly from the feed to the final products or derives from the microbial activity of the rumen and/or from the metabolism of the animal under the effect of specific diets. These compounds can therefore serve as a marker of an animal’s food background [30].

The present study investigated the FA profile of salami made by processing meat from carcasses of different commercial categories, such as grazing or housed young bulls and grazing adult cows of Cinisara breed, under different microbiological conditions due to spontaneous fermentation or the inoculum of a starter culture. This investigation provides additional information to that reported by Gaglio et al. [7] regarding the physicochemical and sensory properties of salami, with the aim to further characterize this product.

## 2. Materials and Methods

### 2.1. Meat and Salami Manufacturing

Carcasses of animals of Cinisara breed, selected on the basis of age and the livestock system (use or non-use of pasture) were used, and in the specific: 

n. 4 grazing young bulls (GB–18 months old), fed pasture-based diets from a 6-month age until slaughtering, with hay and concentrate supplements in the final phase (16–18 months);

n. 4 housed young bulls (HB–18 months old), fed pasture-based diets from a 6-month age, whereas housed and fed a diet of only hay and concentrate in the finishing phase (16–18 months);

n. 4 adult cows at the end of their productive life (AC–10 years old), fed pasture-based diets from 6 months of age until slaughtering, receiving hay and concentrate supplements from the beginning of the reproductive career (24 months).

The animals were slaughtered at an EU-licensed abattoir, according to the standard handling procedures with respect to EU regulations [31] on the protection of animals at the time of slaughter. The carcasses were stored in a cooling room at 4–8 °C for a 7-day ageing period. After, they were dissected (day zero), and the meat from the forequarters of each carcass was minced separately with a 6 mm plate; 50 kg of minced meat of each carcass was used.Each batch of minced meat was supplemented with pork lard (20% *w*/*w*) from Nebrodi Black Pig (Sicilian native pig breed) and other ingredients (sodium chloride, 2.5%; sucrose, dextrose and maltodextrin,0.35%; sodium ascorbate, 0.016%; sodium nitrate, 0.01%; potassium nitrate, 0.051%),obtaining in a blender a raw mixture for each animal (4 for each animal category, 12 total mixtures). Subsequently, each raw mixture was separated into two batches; one part fermented spontaneously, while the other one was inoculated with freeze-dried cultures, containing *Staphylococcus xylosus* and *Pedioccocuspentosaceus* (Tec-AL. s.r.l., Traversetolo, Italy; final concentration of approximately 107 CFU g^−1^). 

The meat was separately stuffed into natural casings. Each salami was formed at approximately 35 cm in length, 7 cm in diameter and an initial weight of about 1.2 kg. Dissection, mincing, blending and stuffing operations were performed on the same day for all carcasses. Afterwards, the salamis were hung for 3 days at 20 °C and ambient RH, and then transferred to cold rooms with controlled temperature and RH following the protocol reported by Gaglio et al. [7], reaching a final weight of about 0.7 kg. In brief, the ripening protocol was as follows: day 1, 20–22 °C and 62–72% RH; day 2, 19–21 °C and 64–74% RH; day 3, 18–20 °C and 66–76% RH; day 4, 17–19 °C and 68–78% RH; day 5, 16–18 °C and 70–80% RH; day 6, 15–17 °C and 72–82% RH; day 7, 14–16 °C and 74–84% RH; day 8, 13–15 °C and 76–86% RH; days 9–10, 12–14 °C and 78–88% RH; days 11–55, 11–13 °C and 80–90% RH. All salami were made at the “Lipari salami factory” in Alcamo (Sicily, Italy).

### 2.2. Sampling

For each carcass, a sample of minced meat (before the addition of other ingredients) and pork fat was collected.

The salami were sampled at day 0 after mixing all ingredients (raw mixture) (12 samples, 1 for each animal) and at day 45 (end of the maturing process) (12 samples of uninoculated salami and 12 samples of inoculated salami). The samples, placed in sterile vacuum containers, were refrigerated at 8°C for the transfer to laboratory where they were homogenized (2 min at maximum speed) by stomacher (LAB Blender 400, Seward Medical, London, UK) and then freeze-dried for successive analysis as described by Alabiso et al. [10] (SCANVAC Coolsafe 55-9, Labogene Aps, Lynge, Denmark).

### 2.3. Fatty Acids Composition

Fatty acids (FA) were extracted according to the method developed by O’Fallon et al. [32]. The total FA quantification was performed using C23:0 (Sigma-Aldrich, Darmstadt, Germany) as internal standard (0.5 mg/g freeze-dried sample). Each sample (1 µL) was injected by autosampler into an HP 6890 gas chromatography system equipped with a flameionization detector (Agilent Technologies Inc., Santa Clara, CA, USA). For separation of FA methyl esters from each sample, a capillary column (100 m length, 0.25 mm i.d., 0.25 µm; CP-Sil 88; Chrompack, Middelburg, the Netherlands) was used. Temperatures of the injector and the detector were kept at 255 °C and 250 °C, respectively, using H_2_ flow of 40 mL/min, air flow of 400 mL/min and He flow of 45 mL/min. The oven temperature was held for 1 min at 70 °C, increased at 5 °C/min to 100 °C, held for 2 min, increased at 10 °C/min to 175 °C, held for 40 min and finally increased at 5 °C/min to 225 °C, held for 45 min. The carrier gas was He, used with a head pressure of 158.6 kPa and a flow rate of 0.7 mL/min (linear velocity of 14 cm/s). The identification of each FA was performed as described by Alabiso et al. [4].

The health-promoting index (HPI) was calculated as suggested by Ashkezary et al. [33]: (*n-3* PUFA + *n-6* PUFA + MUFA)/(C12:0 + (4 × C14:0) + C16:0). The thrombogenic index (TI) was calculated according to Ulbricht and Southgate [34] as follows: TI = (C14:0 + C16:0 + C18:0)/((0.5 × ΣMUFA) + (0.5 × ΣPUFA *n-6*) + (3 × ΣPUFA *n-3*) + (*n-3*/*n-6*)).

### 2.4. Statistical and Explorative Multivariate Analysis

Data were statistically processed using the SAS 9.2 software [35]. FA composition was analyzed according to a MIXED model including the fixed effects of animal category (A, with three levels: GB, HB and AC), product (P, with three levels: raw mixture RM, inoculated salami IS and uninoculated salami US) and the animal within category as a random effect. The interactions between the effects A × P was removed from the model since it was always not significant. Tukey’s test was used to compare means when the effects were significant (*p* < 0.05).

The principal components analysis (PCA), performed using the PRINCOMP SAS procedure, was based on FA composition in order to assess its specific contribution in explaining the differences among salami type, due to the different animal category. The variables used in the analysis were identified on the basis of a stepwise selection using the STEPDISC SAS procedure, after they were standardized by multiplying them by the inverse of standard deviation (1/SD). The number of main components was selected according to Kaiser’s criterion and only those with Eigen values above 1.00 were retained.

## 3. Results and Discussion

### 3.1. Fatty Acids Composition

Table 1 shows the FA composition of meat of each animal category and the pork lard. AC is characterized by a higher percentage content of monounsaturated fatty acids (MUFA) and a lower presence of polyunsaturated fatty acids (PUFA) compared to the levels found in GB, similar to what was found in bresaola obtained from cows at the end of their career [4]. Intermediate features were found in HB.

Pork lard showed a high MUFA content, consisting of 87% of oleic acid (OA, C18:1 *n-9*), and a low PUFA content possibly related to direct deposition of MUFA from acorns present in the diet to the fat depots. The FA composition of pork lard is comparable to that found by Pugliese et al. [36] in outdoor rearing system. The FA composition of pork lard used suggests that it derived from animals that evidently were reared outdoors with a feeding integration to the natural pasture, according to the farming system typically used for the Nebrodi Black Pig.

In general, the processed products show fat quantity and profile depending on the ingredients of the mixture. In this study, the same receipt was used for all products, changing only the kind of meat. Therefore, the differences in fat content and FA composition should be mainly attributable to beef rather than to lard and other ingredients.

Table 2 shows FA profile in relation to the animal category and the product type.

The total FA content (Table 2) was higher in AC than in GB and HB (*p* < 0.05), confirming that meat of adult cows is characterized by a major fat content compared to young animals [5] and that fat and proportions of FA change depending on the age of the animal and its diet [4].

The saturated fatty acids (SFA) (Table 2) did not significantly change among animal categories, while MUFA were higher in AC (*p* ≤ 0.05) compared to GB. HB showed intermediate values (differences not statistically significant). PUFA were higher in GB than in HB, which yet registered higher values compared with AC (*p* ≤ 0.01). 

The animal category influenced the *n-6* and *n-3* contents, the PUFA/SFA and *n-6*/*n-3* ratio, as well as HPI (Table 2). GB showed higher values of both *n-6* (*p* ≤ 0.01) and *n-3* (*p* ≤ 0.05) compared to AC and HB, and higher HPI (*p* ≤ 0.05) compared to AC. The ratio PUFA/SFA was lower in AC compared to GB and HB (*p* ≤ 0.05), while the ratio *n-6*/*n-3* was higher in HB compared to AC (*p* ≤ 0.05). The FAO/WHO report indicates an optimal *n-6*/*n-3* ratio of 4.5–5 [37]. A diet contrasting various “lifestyle diseases”, such as coronary heart diseases and cancers, requires a ratio of PUFA/SFA above about 0.45 and a ratio of *n-6*/*n-3* below 4.0 [38]. Therefore, AC show an unfavorable PUFA/SFA ratio compared to GB and HB. However, despite the lower PUFA content, AC showed a health favorable *n-6*/*n-3* ratio, especially due to the lower linoleic acid (LA) level.

Table 3 shows the SFA composition of raw mixtures and ripened salami. Significant differences were found for palmitic acid (C16:0), higher for HB and AC compared to GB (*p* ≤ 0.01), for C15:0 and C17:0, lower in GB, and for stearic acid (C18:0) higher in GB compared to AC (*p* ≤ 0.05), as found in bresaola by Alabiso et al. [4]. The effect of the product type emerged for C17:0, lower in the raw mixtures than in salami.

Table 4 shows the MUFA composition of raw mixtures and ripened salami. In particular, the C14:1 and especially OA were higher in AC than in GB (*p* ≤ 0.05), while HB showed intermediate values (not statistically significant); as in bresaola, OA was the most representative of the MUFA and its content was influenced by the age of the animals [4,29]. The raw mixtures showed the lower level of C17:1.

Table 5 shows the PUFA composition of raw mixtures and ripened salami. In all animal categories, the most represented among PUFA was linoleic acid (LA, 18:2 *n-6*), and was lower in AC compared to HB and GB (*p* < 0.01). In accordance with Wood et al. [29], the LA content was negatively related to the trend of salami fat. In fact, total FA content (Table 2) was higher in AC than in HB and GB. Furthermore, the arachidonic acid (AA, C20:4 *n-6*), derived from LA through the action of Δ5 and Δ6 desaturase enzymes and elongase [4], was on average lower in AC compared to HB and GB (*p* < 0.05), similar to its precursor. Moreover, eicosapentaenoic (EPA, C20:5 *n-3*) and docosapentaenoic (DPA, C22:5 *n-3*) acids were on average higher in GB than HB and AC (*p* < 0.05 and *p* < 0.01, respectively). Feeding systems based on grazing have been known to have a positive effect on PUFA content of beef, and in general, on products obtained from ruminants [39,40,41]. Indeed, the use of grazing until slaughter resulted in a higher PUFA content of GB compared to HB.AC, despite being grazed until slaughter, showed lower PUFA concentrations than GB.These trends can be linked to the older age of AC, confirming the results obtained in bresaola, in which beef had higher concentrations of fat and lower PUFA levels than those of younger subjects, fed under the same conditions. Analogous differences were found between HB and GB which differed in the feeding pattern [4].

The overall concentration of rumenic acid (RA), the main of CLA isomers, in ripened salami was higher in AC (*p* < 0.05), although the differences between the commercial categories of salami were smaller with respect to those found in bresaola [4], probably due to the mitigating effect of pork lard addition.

Since the addition of pork lard did not induce any positive effects due to the diet and farming system of pigs, the FA composition was better than that found on salami produced with only Nebrodi Black Pig [42].

The fermentation processes in both inoculated and uninoculated salami did not show appreciable differences in FA composition at the end of the maturation. A significant increase in the raw mixture in comparison with the ripened salami was found only for C17:0, C17:1 and others, C18:2 (*p* < 0.05). As found in Gaglio et al. [7], the microbiological evolution may have been comparable between inoculated and uninoculated samples, especially with regard toits role in modifying the FA profile, probably as a consequence of microbial contamination of raw meat during slaughter and subsequent processing stages.

### 3.2. Multivariate Analysis

**Figure 1:**
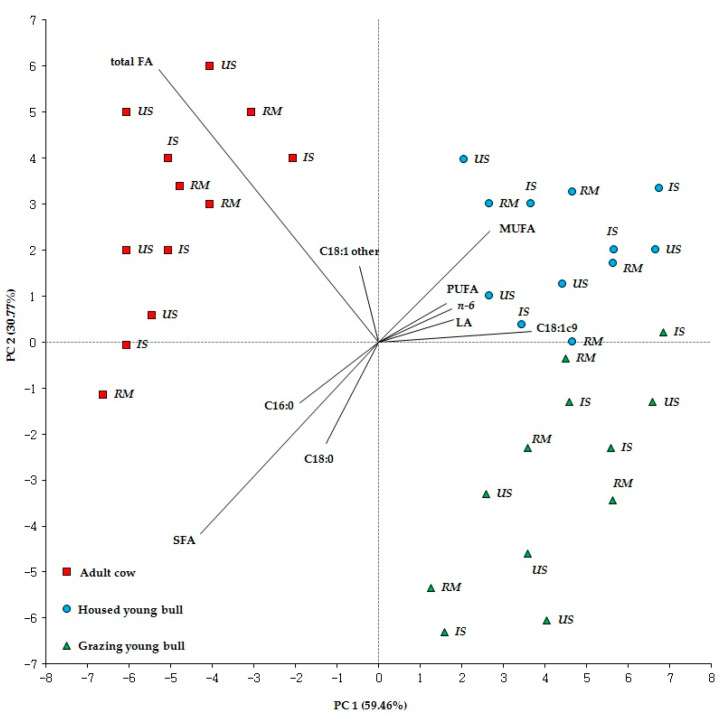
Score plot and loading plot from principal component analysis (PCA) of raw mixture and ripened salami. Abbreviations: RM = raw mixture; IS = inoculated salami; US = uninoculated salami; SFA = saturated fatty acids; MUFA = monounsaturated fatty acids; PUFA = polyunsaturated fatty acids; LA = linoleic acid.

The plot generated by PCA is shown in Figure 1, where the length of each vector measures the contribution of each variable to the main components.

The first two principal components accounted for 90.23% of the total variance.With 59.46% of the total variance, the first principal component was able to discriminate the AC salami from those of GB and HB on the basis of the main contributions of total FA, SFA, C18:1*n-9* and MUFA.Instead, with 30.77% of the total variance, the second main component was able to discriminate GB salami from HB salami, especially for the contribution of total FA, SFA, C18:0, MUFA and C16:0. Overall, the animal category, which associates the effect of age and feeding system, influenced the qualitative traits of salami more markedly than the inoculated starter culture, since the effect of the inoculation did not allow a clear distinction of the salami within the animal category.

## 4. Conclusions

The origin of meat, with regard to the animal category, differing for age and feeding system, influenced the FA composition of salami. The addition of pork lard reduced the initial differences between the animal categories.

In bulls, the use of pasture up to slaughter improved the FA profile, determining an increase in *n-3* PUFA content. Adult cows, despite benefiting from the pasture until slaughter, showed lower PUFA contents than young bulls, due to the higher fat content. However, the lower LA level resulted in a more favorable *n-6*/*n-3* ratio in AC than HB. The higher age also determined an increase of OA in AC.

On the basis of these results, salami made with Cinisara meat could be an alternative product, even if the addition of pork lard mitigated the favorable effect of the livestock system based on grazing in increasing the PUFA content and thus in improving the FA profile.

In salami, fermentation processes and the addition of pork lard would allow one to appreciate only significant differences linked to the animal categories. Further studies should be conducted in which salami is made only with beef, considering also the consumers who do not eat pork.

## Figures and Tables

**Table 1:** Fatty acids profile (% of total FA) and health indexes of fresh meat and fresh pork lard.

	Animal Category (A) ^a^			
	GB	HB	AC	PL
Fat	3.30	4.65	8.80	76.12
Total FA,% DM ^b^	2.94	4.19	7.92	68.52
Others SFA ^c^	0.71	1.43	1.87	n.d.
C14:0	2.32	2.02	2.23	n.d.
C14:1	0.03	0.08	0.06	2.12
C15:0	1.89	1.30	1.80	0.05
C16:0	16.06	19.24	20.84	26.18
C16:1	2.24	2.4	1.91	3.51
C17:0	1.57	1.13	1.42	0.39
C17:1	0.50	0.76	1,00	0.16
C18:0	19.19	18.55	16.91	9.28
C18:1 c9	17.42	24.38	35.51	43.29
C18:1 t11	1.14	1.23	1.59	n.d.
Other C18:1	1.41	1.51	1.76	0.67
Other C18:2	0.32	0.24	0.64	0.07
C18:2 *n-6* LA	19.83	13.21	4.07	11.27
CLA C18:2 c9t11 RA	0.16	0.21	0.22	0.12
Other CLA isomers	0.09	0.03	0.07	0.05
C18:3 *n-3* ALA	2.35	1.25	1.22	0.61
C18:3 *n-6* GLA	0.16	0.18	0.19	0.17
C20:0	0.16	0.12	0.11	0.10
C20:2 *n-6*	0.15	0.17	0.13	0.04
C20:3 *n-6*	0.42	0.40	0.43	0.08
C20:4 *n-6* AA	6.97	5.83	2.61	0.93
C20:5 *n-3* EPA	0.92	0.59	0.41	0.28
C22:0	1.13	1.08	1.01	0.03
C22:2 *n-6*	0.22	0.15	0.05	n.d.
C22:4 *n-6*	0.37	0.32	0.21	n.d.
C22:5 *n*3 DPA	2.03	1.49	0.85	n.d.
SFA	43.03	44.87	46.19	36.03
MUFA ^d^	22.74	30.36	41.83	49.75
PUFA ^e^	33.99	24.07	11.10	13.62
MUFA/SFA	0.53	0.68	0.91	1.38
PUFA/SFA	0.79	0.54	0.24	0.38
*n-6*	28.12	20.26	7.69	12.49
*n-3*	5.30	3.33	2.48	0.89
*n-6*/*n-3*	5.31	6.08	3.10	14.03
HPI ^f^	2.23	1.98	1.76	2.42
TI ^g^	0.90	1.09	1.22	1.04

The results indicate mean values of three measurements performed on each of the four carcasses for animal category and for each sample of lard. ^a^ Animal category: GB = grazing young bull; HB = housed young bull; AC = adult cow; PL = pork lard. ^b^ DM = dry matter; ^c^ SFA = saturated fatty acids; ^d^ MUFA = monounsaturated fatty acids; ^e^ PUFA = polyunsaturated fatty acids; ^f^ HPI = health promoting index; ^g^ TI = thrombogenic index.

**Table 2:** Effects of animal category and product on fatty acids profile (% of total FA) and health indexes of salami fat.

	Product (P) ^b^		Animal Category (A) ^a^			SEM ^h^	Significance	
			GB	HB	AC		A	P
Total FA,% DM	RM	34.86	30.76	33.40	40.40	1.569	0.042	0.538
	IS	35.79	31.17	33.79	42.42			
	US	36.86	34.24	35.11	41.23			
	Total		31.38 ^b^	34.10 ^b^	41.35 ^a^			
SFA ^c^	RM	39.76	39.03	39.39	40.85	2.872	0.650	0.363
	IS	39.98	38.92	39.85	41.17			
	US	39.80	38.68	40.26	40.46			
	Total		38.88	39.83	40.82			
MUFA ^d^	RM	39.60	34.01	39.33	45.45	2.085	0.042	0.469
	IS	40.96	35.85	40.27	46.75			
	US	40.85	35.66	40.12	46.77			
	Total		35.17 ^b^	39.90 ^a, b^	46.32 ^a^			
PUFA ^e^	RM	20.18	26.42	21.08	13.04	0.989	0.008	0.780
	IS	18.84	25.16	19.30	12.07			
	US	18.86	25.18	19.48	11.93			
	Total		25.59 ^a^	19.95 ^b^	12.35 ^c^			
MUFA/SFA	RM	0.99	0.87	1.00	1.11	0.196	0.407	0.358
	IS	1.02	0.92	1.01	1.14			
	US	1.03	0.93	1.00	1.15			
	Total		0.91	1.00	1.14			
PUFA/SFA	RM	0.51	0.68	0.53	0.32	0.146	0.031	0.376
	IS	0.48	0.65	0.49	0.29			
	US	0.47	0.65	0.48	0.29			
	Total		0.66 ^a^	0.51 ^a^	0.30 ^b^			
*n-6*	RM	17.33	22.78	18.50	10.77	0.997	0.007	0.258
	IS	15.98	21.48	16.63	9.79			
	US	15.91	21.40	16.75	9.57			
	Total		21.88 ^a^	17.29 ^b^	10.04 ^c^			
*n-3*	RM	2.62	3.51	2.39	1.97	0.095	0.008	0.668
	IS	2.53	3.45	2.30	1.84			
	US	2.61	3.57	2.40	1.86			
	Total		3.51 ^a^	2.36 ^b^	1.89 ^b^			
*n-6*/*n-3*	RM	6.57	6.49	7.74	5.47	0.752	0.031	0.529
	IS	6.26	6.23	7.23	5.32			
	US	6.04	5.99	6.98	5.15			
	Total		6.24 ^a, b^	7.31 ^a^	5.31 ^b^			
HPI ^f^	RM	2.05	2.21	2.06	1.87	0.054	0.041	0.843
	IS	2.03	2.35	1.93	1.82			
	US	2.04	2.29	2.01	1.81			
	Total		2.28 ^a^	2.00 ^a, b^	1.83 ^b^			
TI ^g^	RM	1.08	0.98	1.10	1.16	0.096	0.576	0.809
	IS	1.06	0.97	1.13	1.17			
	US	1.08	0.97	1.12	1.18			
	Total		0.98	1.12	1.17			

The results indicate mean values of three measurements performed on each trial of each carcass. ^a^ Animal category: GB = grazing young bull; HB = housed young bull; AC = adult cow. ^b^ Product: RM= raw mixture (day 0), IS= inoculated salami (day 45), US = uninoculated salami (day 45).^c^ SFA = saturated fatty acids; ^d^ MUFA = monounsaturated fatty acids; ^e^ PUFA = polyunsaturated fatty acids; ^f^ HPI = health promoting index; ^g^ TI = thrombogenic index. ^h^ SEM= standard error of the means. The interactions among the effects were removed from the model because they were not significant (*p* > 0.05). Different letters (^a, b, c^) on horizontal rows indicate *p* < 0.05.

**Table 3:** Effects of animal category and product on saturated fatty acids (% of total FA) of salami fat.

	Product (P) ^b^		Animal Category (A) ^a^			SEM ^d^	Significance	
			GB	HB	AC		A	P
Others SFA ^c^	RM	0.77	0.23	0.66	1.40	0.328	0.419	0.685
	IS	0.79	0.83	0.3	1.22			
	US	0.54	0.24	0.79	0.61			
	Total		0.44	0.58	1.08			
C14:0	RM	1.62	1.64	1.51	1.71	0.136	0.089	0.653
	IS	1.71	1.48	1.84	1.81			
	US	1.64	1.5	1.55	1.89			
	Total		1.54	1.63	1.80			
C15:0	RM	0.24	0.20	0.16	0.37	0.071	0.021	0.752
	IS	0.31	0.23	0.33	0.39			
	US	0.30	0.15	0.24	0.51			
	Total		0.19 ^b^	0.25 ^b^	0.42 ^a^			
C16:0	RM	22.81	20.80	23.25	24.39	0.983	0.009	0.462
	IS	22.80	20.04	23.39	24.98			
	US	22.89	20.52	23.36	24.78			
	Total		20.50 ^b^	23.35 ^a^	26.71 ^a^			
C17:0	RM	0.46 ^b^	0.47	0.35	0.58	0.091	0.029	0.019
	IS	0.65 ^a^	0.53	0.67	0.74			
	US	0.65 ^a^	0.43	0.56	0.94			
	Total		0.48 ^b^	0.53 ^b^	0.76 ^a^			
C18:0	RM	13.01	14.82	12.61	11.60	1.084	0.026	0.291
	IS	12.97	15.06	12.55	11.29			
	US	12.74	14.18	12.97	11.07			
	Total		14.67 ^a^	12.71 ^a, b^	11.32 ^b^			
C20:0	RM	0.10	0.10	0.10	0.10	0.019	0.299	0.738
	IS	0.08	0.10	0.09	0.08			
	US	0.10	0.09	0.10	0.10			
	Total		0.10	0.10	0.10			
C22:0	RM	0.73	0.77	0.75	0.70	0.080	0.938	0.093
	IS	0.66	0.65	0.68	0.66			
	US	0.67	0.76	0.69	0.56			
	Total		0.72	0.71	0.65			

The results indicate mean values of three measurements performed on each trial of each carcass. ^a^ Animal category: GB = grazing young bull; HB = housed young bull; AC = adult cow. ^b^ Product: RM= raw mixture (day 0), IS= inoculated salami (day 45), US = uninoculated salami (day 45). ^c^ SFA = saturated fatty acids. ^d^ SEM= standard error of the means. The interactions among the effects were removed from the model because they were not significant (*p*>0.05). Different letters (^a, b^) on horizontal and vertical rows indicate *p* < 0.05.

**Table 4:** Effects of animal category and product on monounsaturated fatty acids (% of total FA) of salami fat.

	Product (P) ^b^		Animal Category (A) ^a^			SEM ^c^	Significance	
			GB	HB	AC		A	P
C14:1	RM	0.08	0.07	0.06	0.12	0.025	0.006	0.997
	IS	0.09	0.04	0.11	0.14			
	US	0.10	0.03	0.08	0.18			
	Total		0.04 ^b^	0.08 ^b^	0.18 ^a^			
C16:1	RM	2.41	2.55	2.24	2.46	0.203	0.066	0.062
	IS	2.60	2.21	2.70	2.81			
	US	2.67	2.47	2.53	3.01			
	Total		2.43	2.49	2.76			
C17:1	RM	0.27 ^b^	0.27	0.25	0.29	0.022	0.218	0.041
	IS	0.36 ^a^	0.32	0.37	0.43			
	US	0.36 ^a^	0.30	0.36	0.53			
	Total		0.30	0.33	0.37			
C18:1 c9 OA	RM	34.58	29.33	34.46	39.94	2.440	0.031	0.598
	IS	34.68	30.41	33.83	39.80			
	US	34.38	30.16	33.41	39.57			
	Total		32.80 ^b^	37.56 ^a, b^	40.44 ^a^			
Other C18:1	RM	2.25	1.79	2.32	2.64	0.764	0.241	0.439
	IS	3.90	3.87	4.26	3.57			
	US	3.64	3.70	3.74	3.48			
	Total		2.79	3.44	3.23			

The results indicate mean values of three measurements performed on each trial of each carcass. ^a^ Animal category: GB = grazing young bull; HB = housed young bull; AC = adult cow. ^b^ Product: RM= raw mixture (day 0), IS= inoculated salami (day 45), US = uninoculated salami (day 45). ^c^ SEM= standard error of the means. The interactions among the effects were removed from the model because not significant (*p* > 0.05). Different letters (^a, b^) on horizontal and vertical rows indicate *p* < 0.05.

**Table 5:** Effects of animal category and product on polyunsaturated fatty acids (% of total FA) of salami fat.

	Product (P) ^b^		Animal Categories (A) ^a^			SEM ^c^	Significance	
			GB	HB	AC		A	P
Other C18:2	RM	0.06 ^b^	0.03	0.03	0.12	0.024	0.027	0.011
	IS	0.16 ^a^	0.08	0.17	0.22			
	US	0.15 ^a^	0.06	0.14	0.26			
	Total		0.05 ^c^	0.11 ^b^	0.20 ^a^			
C18:2 *n-6* LA	RM	13.76	18.29	14.47	8.52	0.704	0.015	0.281
	IS	12.71	17.69	12.87	7.56			
	US	12.65	17.61	13.02	7.33			
	Total		17.86 ^a^	13.45 ^b^	7.80 ^c^			
CLA C18:2 c9t11 RA	RM	0.11	0.12	0.10	0.12	0.013	0.039	0.256
	IS	0.11	0.11	0.07	0.14			
	US	0.11	0.10	0.08	0.15			
	Total		0.11 ^b^	0.11 ^b^	0.14 ^a^			
Other CLA isomers	RM	0.04	0.08	0.04	0.06	0.015	0.483	0.111
	IS	0.09	0.08	0.09	0.08			
	US	0.07	0.07	0.09	0.09			
	Total		0.07	0.07	0.07			
C18:3 *n-3* ALA	RM	1.42	1.78	1.24	1.24	0.073	0.059	0.361
	IS	1.33	1.73	1.12	1.14			
	US	1.37	1.79	1.18	1.15			
	Total		1.77	1.18	1.18			
C18:3 *n-6* GLA	RM	0.16	0.15	0.16	0.15	0.017	0.118	0.128
	IS	0.18	0.21	0.17	0.17			
	US	0.18	0.20	0.17	0.16			
	Total		0.19	0.17	0.16			
C20:2 *n-6*	RM	0.10	0.11	0.10	0.09	0.027	0.420	0.230
	IS	0.12	0.13	0.15	0.08			
	US	0.10	0.10	0.09	0.11			
	Total		0.11	0.11	0.09			
C20:3 *n-6*	RM	0.06	0.07	0.06	0.05	0.012	0.110	0.273
	IS	0.07	0.06	0.06	0.09			
	US	0.02	0.01	0.01	0.04			
	Total		0.05	0.04	0.06			
C20:4 *n-6* AA	RM	3.03	3.86	3.42	1.81	0.013	0.041	0.651
	IS	2.67	3.13	3.11	1.77			
	US	2.73	3.22	3.19	1.78			
	Total		3.40 ^a^	3.24 ^a^	1.79 ^b^			
C20:5 *n-3* EPA	RM	0.48	0.69	0.41	0.35	0.031	0.039	0.628
	IS	0.48	0.67	0.43	0.34			
	US	0.51	0.72	0.47	0.34			
	Total		0.69 ^a^	0.44 ^a, b^	0.34 ^b^			
C22:2 *n-6*	RM	0.11	0.15	0.14	0.05	0.003	0.036	0.361
	IS	0.10	0.13	0.14	0.03			
	US	0.10	0.13	0.13	0.03			
	Total		0.14 ^a^	0.14 ^a^	0.04 ^b^			
C22:4 *n-6*	RM	0.13	0.15	0.15	0.10	0.009	0.305	0.306
	IS	0.12	0.13	0.13	0.09			
	US	0.13	0.13	0.14	0.12			
	Total		0.14	0.14	0.10			
C22:5 *n-3* DPA	RM	0.72	1.04	0.74	0.38	0.007	0.009	0.242
	IS	0.72	1.05	0.75	0.36			
	US	0.73	1.06	0.75	0.37			
	Total		1.05 ^a^	0.75 ^b^	0.37 ^c^			

The results indicate mean values of three measurements performed on each trial of each carcass. ^a^ Animal category: GB = grazing young bull; HB = housed young bull; AC = adult cow. ^b^ Product: RM= raw mixture (day 0), IS = inoculated salami (day 45), US = uninoculated salami (day 45). ^c^ SEM = standard error of the means. The interactions among the effects were removed from the model because they were not significant (*p*>0.05). Different letters (^a, b^) on horizontal and vertical rows indicate *p* < 0.05. LA = linoleic acid; ALA = α-linolenic acid; RA = rumenic acid; CLA = conjugated linoleic acid; AA = arachidonic acid; EPA = eicosapentaenoic acid; DPA = docosapentaenoic acid.

## Data Availability

All data included in this study are available upon request by contacting the corresponding author.
